# Francis Thompson: Medical Student and Poet

**Published:** 1922-01

**Authors:** 


### THE REVIEW OF THE MONTH.

### FRANCIS THOMPSON: MEDICAL STUDENT AND POET.

THE son of a medical practitioner, Francis Thompson, who was born "in 1858 or 1859 (I never could rememoer which)," after leaving Ushaw College, Durham, in July, 1877, became a medical student at Owen's College, Manchester. We do not know much more of his student days than of those of Keats, but whereas Keats qualified, Thompson never sat; at least, he never passed his medical examinations. Under the pressure of his family, he spent six years at this uncongenial task. He was a lax attendant at lectures, constitutionally absent-minded, solitary, reserved; and his biographer, Mr. Everard Meynell, records the poet's own impression of his medical training: "I hated my scientific and medical studies, and learned them badly. Now, even that bad and reluctant knowledge has grown priceless to me." During these years "he was much at the Old Trafford ground," and had the pathetic respect of the man of letters for cricket, reeling off the names of the great players and recounting the great matches with the ardour of the unathletic sportsman. His eager and undoubted knowledge of the game begot no poems of note, but, as we shall see, his reluctant knowledge of science engendered more than one splendid phrase in his writings.

In 1879 he had a serious illness and, according to his biographer, it was probably at this time that Thompson first tasted laudanum. It was at this time, too, that his mother presented him with a copy of De Quincey's "Confessions of an English Opium-Eater." The parallel between the lives of De Quincey and Thompson (not to mention Coleridge), in their flights from Manchester, their wanderings in London, their beautiful friendships with a woman of the streets, their addiction to opium, has been drawn too often to need further repetition. But Mr. Meynell adds an interesting touch. "Opium" (he says) "was in the air of Manchester, the cotton-spinners being much addicted to its use." Of the effect of the drug upon Thompson's constitution, his biographer observes: "It staved off the assaults of tuberculosis [of which the poet died in 1907]; it gave him the wavering strength that made life just possible for him, whether on the streets or through all those other distresses and discomforts that it was his character deeply to resent but not to remove by any normal courses." This opinion Thompson himself corroborates; "in one of his own most rare allusions to it," he speaks of "opium, the saving of my life."

At the end of two years' study, he said to his family, "I have not passed," and in 1882 this terse formula had to be repeated. "During two more years of preparation he read less and less at home"; and hours which he affirmed to have been spent on "extra instruction" were proved in time to have been passed in the home of a musician, listening to Berlioz and Beethoven. His third attempt upon the examiners was to be made at Glasgow. It would not have been made at all if Thompson had not been afraid to dispute his father's determination. Thompson has been blamed for his want of courage. Surely the blame must be divided in equal shares. Children are endowed by nature with an affection for their parents which allows an ample margin for parental mistakes. But when a child does not dare to confide in its parent, the latter receives a proof of failure which no argument can whittle away. Children will be friends with their elders just so far as their elders will allow. Such a friendship makes large demands upon the elder, but an elder is supposed to be equal to demands, and, where children are concerned, to go more than half-way in meeting them. When Francis Thompson failed at Glasgow, the father was so incensed that he apprenticed his son to a surgical instrument-maker, with whom the apprentice remained for two weeks. Then a new opening was found for him—purveyor of an encyclopædia! It is so natural to sympathise with parents whose children upset their cherished schemes, that we cannot help smiling when they make themselves figures of comedy. Not the least use of such smiles is to help us to beware of similar behaviour. After spending two months upon the perusal of the encyclopædia, Thompson "discarded it, unsold." On Monday, November 9, 1885, he left a note saying that he had gone to London. He went first to Manchester, where he remained a week. In response to his request, the fare to London had been sent from home. He approached London without ambition, in truth, without hope. He was to find there the courage to endure which he had failed to find in the presence of his father.

The rest of his life, after medicine ceased to influence it, can be briefly sketched. For some years he lived the life of a complete outcast, avoiding even the few friends within his reach, and at last not calling at the place where the weekly pittance awaited him. He was stupefied with want. His chief relief was opium. He learned to know the bitter life of the streets, to sleep in shelters or on the Embankment. He carried sacks of books, and, later, of boots, for a friendly salesman in Panton Street. He ran after cabs; he sold matches, and newspapers. He held horses, and scanned, twice successfully, the pavement for pennies; two, indeed, were sovereigns. "Only once did anyone try to cheat me." But he discovered, "as month succeeded month, that death is surprisingly slow on a shilling a day." The Christmas of 1886 he spent at home, and in the reserve* which was characteristic of his family the veil was hardly lifted. In January, 1887, he left Panton Street, and the next month, probably after the arrival of a few shillings from home, he was enough himself to piece together the paper on "Paganism Old and New" which eventually introduced his name to Mr. Wilfred Meynell, who then edited "*Merry England*." Some

---

I thank the once accursed star
Which did me teach
To make of Silence my familiar. . . .
—From *Sister Songs*.

**The Review of the Month—(*cont.*).**

months later, thanks to the persistent efforts of the editor, he and his contributor met:—

"A waif of a man came in. No such figure had been looked for; more ragged and unkempt than the average beggar, with no shirt beneath his coat and bare feet in broken shoes." With difficulty he was persuaded to call again. With equal difficulty he was eventually rescued. The rest of his life was the nursling of his friends, and its fruit the poems which have made them both famous. Suffice it to say that "after a course of medical treatment"—("He will not live, and you hasten his death by denying his whims and opium" was the first prognosis)—he was sent to Storrington, the Sussex village where his poetic genius began to unfold at its own leisure. As he resisted the habit, so, we are told, his poetic faculty vivified. At midsummer, 1889 "The Ode to the Setting Sun" was written. Soon afterwards he began his essay on Shelley. It was a solitary attempt to please the "Dublin Review," which, at that time, was too much in ecclesiastical apron strings not to look askance at humaner letters. The essay, at first refused, was published in the Review after Thompson's death, and became formally part of English letters with its subsequent publication in a sl'm volume of 76 pages.

This is one of the most lyrical of English essays, too lyrical perhaps for true prose. This said, and the preliminary cough of the opening paragraphs allowed for, it detains us, here and now, chiefly for a single expression, which recalls the experiment that every medical student is invited to observe at his first lecture in chemistry. Speaking of Shelley's power of "apprehending everything in figure," Thompson says: "The dimmest-sparked chip of a conception blazes and scintillates in the oxygen of his mind." This power, "so almost preternaturally developed in no other poet," enables him "to condense the most hydrogenic abstraction. . Science can now educe threads of such exquisite tenuity that only the feet of the tiniest infant-spiders can ascend them; but up the filmiest insubstantiality Shelley runs with agile ease." Again, "Poetry is a thermometer: by taking its average height you can estimate the normal temperature of its writer's mind." These are pleasant echoes from the dusty lecture-hall at Manchester. A poet's love of words can gratify itself in many ways; but, whether he turn, like Petronius, to the racy idiom of the Suburra, or like Shakespeare, in Dr. Johnson's words, "sometimes to the sports of the field, and sometimes to the manufactures of the shop," it is always the technical vocabulary which lures him. The harness-room and the laboratory, the cloister and Doctors' Commons are equally rich in proper speech, which defies every influence except that of elementary education. The shop does not provide Thompson, so far as we recall, with any figures. The law and the laboratory do. For the sports of the field his verse goes to falconry and not to cricket. He speaks of the heart arrassed in purple like the house of Kings, and the well-known dissertation in "Sister Songs" upon the sex which is latent in children is worth exploring, not only by lovers of literature, but by their new friends of the school of Freud and Jung.

In verse his masterpiece is, of course, "The Hound of Heaven," a magnificent ode marred only by a self-conscious oxymoron of three lines in the second stanza. This ode displays all his qualities; his exuberance of fancy, his mystical experience, his learned fecundity of language, his literary assimilations, his rich quarry of words. The sense of the chase has never been expressed better than in the line

Fear wist not to evade as love wist to pursue.

Thompson's language gives an added glow to heat, a richer tone to colour, a new wind-swiftness to speed. For example—

Stay is heat's cradle, it is rocked therein
And by check's hand is burnished into light.

Or

Yea, and His mercy, I do think it well,
Is flashed back from the brazen gates of Hell.

Again, of colour

Every wave upon the harbour-bars
A beaten yolk of stars.

and

The butterfly sunset claps its wings.

and this picture of the creation of light—

When, like the back of a gold-mailèd saurian
Heaving its slow length from Nilotic slime,
The first long gleaming fissure runs aurorian
Athwart the yet dun firmament of prime.

Science is a seam rich in opulent words; and among words, perhaps because nouns appear to have been the oldest words, and of nouns proper names are the dearest, names of places have stirred the human imagination more than any others. When Thompson writes of "rare-gummed Sava and Herbalimar," our thoughts turn at once to our maps and to our Bibles, where such unforgettable syllables as Hamath and Arpad, Hena and Ivah, or Imber, Wessex, and Stow-on-the-Wold evoke landscapes and horizons which make descriptive writing pale and faint. It is less than the truth to say that Thompson's language was archaic. The real truth is that he knit again the jewelled thread which snapped toward the end of the seventeenth century when modern English prose was begotten. After Sir Thomas Browne, Addison, the great Swift himself, resemble skimmed milk, even when we remember the difference of intention. Thompson was returning to a tradition at the point which it attained before it suffered eclipse. The line from "Sister Songs"—"I who can scarcely speak my fellows' speech"—is the utterance of one awake to the modern emasculation of English. He was not, of course, alone in this. For, beneath the material prosperity and solemn ugliness of Victo ian England, an undercurrent of a greater memory flowed. The sense of starvation, subconsciously appealing to an elder tradition, subterraneously flowing beneath the romantic materialism of the Victorian age, produced both in art and letters more than one retrievement of the past. This has continued, to create the new Romanticism of the twentieth century, but not till it shall have re-created our prose will the return of Romance be complete.

**The Review of the Month—(*cont.*).**

Thompson cultivated his prose with the same ardour as his verse. The prose of the poets was a favourite subject for his own essays. Apart from these periodical contributions, since collected in a volume (it must be confessed) of uneven value, only two prose works of his were written. His "life," of St. Ignatius was written by request, and, despite the contrary opinion, the harness jingles unevenly with the movement of the horse's feet. A better, and much briefer, work is the essay "Health and Holiness," to which of all his writings, science, physiology and psychology in particular, contributes most. It is a plea for the better understanding of asceticism, for a return, though this seems to have been imperfectly understood, to the Greek conception. Ascesis originally meant the art of keeping fit. 'Ασκεῖν τὸ σῶμα was to train, not to mortify, the body. Thompson argues that the modern man is more pigeon-livered, more anæmic, than his Elizabethan ancestors, and that their vitality needed a curb which would only macerate our own. He reminds the reader that the body has terrible revenges for those who abuse it, whether from pious or merely self-indulgent motives. "We may sin against the body (he says) in other ways than are catalogued in Liguori; and impoverished blood—who knows?—may mean impoverished morals." Elsewhere in the same essay he remarks, "We find our austerities ready made. The East wind has replaced the discipline, dyspepsia the hair-shirt. . . . It grows a vain thing to mortify the appetite—would we had the appetite to mortify. . . . The mediæval men fight amidst the torrid lands of the East jerkined and breeched in iron which it makes us ache to look upon; our men in khaki fall out by hundreds during peace-manœuvres on an English down . . . . We cannot merely ignore the body: it will not be ignored, and has ungovernable avenues of retaliation . . . St. Ignatius, we have said, came to think he had needlessly crippled his body—after all a necessary servant—by the unweighed severity of Manresa. 'I have been too hard on Brother Ass.' He that sins strongly has the stuff of sanctity rather than the languid sinner."

The conclusion is: "To foster the energies of the body, yes; and to foster the energies of the will: that is the crying need of our uncourageous day. The most potent ally of the Church and ultimate stickler for an ascetic religion—Nature. Holiness energises . . . one might almost say it prolongs life."

That is sound medical doctrine, and, though Thompson hardly recognised it, sound Pagan doctrine too. He has been lauded so much that the time is ripe for criticism to do justice to his lapses from literary virtue. Under the overt glance of the ecclesiastical eye, especially in prose, his spontaneity was frozen. He was too fond of inversion and elision, which wear an affected air in our non-inflected tongue. He overdid the repetition of liturgical details: the acolyte becomes a wearisome intruder in his verse. His language was over-weighted. As he himself said of Crashaw: "The marvel of diction becomes even too conscious . . . it is the feat of an amazing gymnast in words rather than of an unpremeditated angel." But when all is said, he recalled our language to its originals; he had the root of the matter in him, and was perhaps, after all, never more splendid than in his simple passages. The poem "In No Strange Land," the refrain in "To Monica Thought Dying," the gallery of portraits in the "Victorian Ode" are authentic utterance. The rich festoons of language, which drape in heavy folds the jewelled thought of the most often quoted pieces, need no allusion at this time. He crushed our language like a grape and it spurted over his page, at his best in more than purple patches. Indeed, the praise which they have received bespeaks a thirst for the rich wine which used to run in English authors. The praise suggests this more than any due recognition of the tradition to which he belonged. Hence its revival, in our illiterate day, seemed at first so monstrous and so astonishing. Go to him, then, for these things and learn the poverty of your own vocabulary! His style should lead you rather to assimilate the thought which runs naturally into such speech, and to measure modern English by its standard, than to gape at the heap of glittering words themselves. You will not let many of these pebbles run through your fingers without chancing on the white, recurrent pearls that they contain: his sense, and embodiment, of the child-like imagination. It was his clue to Shelley. It enabled him to divine the latency of the woman in the girl. If "Health and Holiness" is a tract on psycho-therapy by the man of letters for the man of science, "Sister Songs" casts a beam of light upon the child's psychology. Is it too much to affirm that the poet was indebted to the medical student, and that Owen's College, Manchester, is a stride nearer Parnassus since Thompson worked unwillingly there?

### THE COLLEGE OF NURSING.

Members of the College of Nursing are reminded that the date for receiving the applications for Midwifery Scholarships has been extended to January 1, 1922. The value of the Scholarships is £50, which should fully cover the cost of training. The forms can be obtained from the Secretary, 7, Henrietta Street, Cavendish Square, W. 1.

**Aberdeen Centre.**

Syllabus of lectures, to be held at the Cowdray Club:—

January 4, at 8 p.m., "Method of Procedure at Business Meetings," Mrs. J. C. Glegg.

January 25, at 8 p.m., "The Web of Life," Professor J. Arthur Thomson, M.A., LL.D.

February 8, at 8 p.m., Social Evening.

February 22, at 8 p.m., "Social Aspects of the Health Problem," Miss Nora Milnes, B.Sc., Director of Social Study, Edinburgh University.

March 22, at 8 p.m., "A Field Ambulance in France" (Lantern Illustrations), Lieut.-Colonel Rorie, D.S.O.

**Liverpool Centre.**

January 4, Miss A. W. Parr, B.A., L.R.A.M., at the Royal Infirmary, will lecture on "Some Aspects of Elocution." Lectures for February, March, April are being arranged, and will be notified later. Owing to the increased cost of postage no cards will be sent this session to members to remind them of the meetings, but due notice will be given in the Nursing papers.

**Norfolk and Norwich Centre.**

Postcards will be issued early in the New Year announcing the subjects and dates of forthcoming lectures.

### ROYAL SANITARY INSTITUTE.

The next Congress of the Royal Sanitary Institute will be held at Bournemouth from July 24 to 29, 1922, by invitation of the Mayor and Town Council.

